# Effect of Dietary Olive Cake Supplementation on Performance, Carcass Characteristics, and Meat Quality of Beef Cattle

**DOI:** 10.3390/ani10071176

**Published:** 2020-07-10

**Authors:** Vincenzo Chiofalo, Luigi Liotta, Vittorio Lo Presti, Fabio Gresta, Ambra Rita Di Rosa, Biagina Chiofalo

**Affiliations:** 1 Consortium Research of Meat and Agrifood, 98168 Messina, Italy; 2 Department of Chemical, Biological, Pharmaceutical and Environmental Sciences, University of Messina, 98166 Messina, Italy; 3 Department of Veterinary Sciences, University of Messina, 98168 Messina, Italy; luigi.liotta@unime.it (L.L.); vittorio.lopresti@unime.it (V.L.P.); fgresta@unime.it (F.G.); dirosaa@unime.it (A.R.D.R.); biagina.chiofalo@unime.it (B.C.)

**Keywords:** beef cattle, olive cake, in vivo performance, carcass traits, meat quality, fatty acid profile

## Abstract

**Simple Summary:**

The consumer’s liking of meat is measured in relation to color, intramuscular fat content, healthy composition of fatty acids, tenderness, juiciness, flavor, and aroma; these qualitative characteristics, influencing the consumer’s choice, guide the market whose objective is to provide safe beef with high food characteristics. The use of agro-industrial co-products, containing appreciable amounts of vegetable oils, could be a feasible strategy to influence the quality of meat. In this study, the effect of the partially destoned olive cake supplementation on the performance, carcass characteristics, and meat quality of beef cattle was evaluated. The experiment was carried out on 45 Limousin bulls divided into three homogenous groups, fed with a diet containing 0%, 7.5%, and 15.0% of the olive cake. Results show that the olive cake supplementation influenced the animal performance, increased the tenderness of meat, the intramuscular fat content and unsaturated fatty acids (oleic acid and essential fatty acids), affecting the meat quality indices and suggesting olive cake as a strategy for the sustainability of the animal food chain, rural economies, and environment, providing healthy animal products.

**Abstract:**

Dietary partially destoned olive cake supplementation on performance, carcass traits and meat quality of intensively finished bulls was evaluated. Forty-five Limousin bulls, divided into three homogenous groups, received a diet with no supplementation (Control-CTR), 7.5% (Low Olive Cake-LOC), and 15.0% of olive cake supplementation (High Olive Cake-HOC). The trial was realized for 150 days; all bulls were individually weighed at the beginning, middle, and end of the trial, to calculate the individual average daily gain (ADG). At slaughtering, on each carcass, hot weight was recorded and, after 7 days, the pH and temperature were measured. On *Longissimus lumborum* muscle, color, cooking loss, and shear force of the cooked sample were determined. The chemical composition and the fatty acid content of muscle were determined. Olive cake inclusions (7.5% and 15.0%) increased (*p* < 0.05) the body weight, ADG, slaughter traits and intramuscular fat content and influenced (*p* < 0.05) the quality indices. The 15.0% of the inclusion reduced (*p* < 0.05) the cooking loss and shear force, and increased the unsaturated fatty acid content. The olive cake can be considered as a functional component in beef production and, in substitution to a quote of cereals into the diet of bulls, could be an opportunity to improve agriculture sustainability.

## 1. Introduction

Worldwide, beef ranks third in consumption (6.4 kg per capita) after poultry (14.0 kg per capita) and pork (12.2 kg per capita) [1]. It is estimated that the consumption of beef, which has been growing continuously since 2015 due to the increase in population and household incomes, will reach a value of +8% and +21% in developed and developing countries in 2027 [2]. It is now well known that beef is a food rich in nutrients beneficial for human health. The acceptance index of meat is measured in relation to color, intramuscular fat content, healthy fatty acids, tenderness, juiciness, flavor, and aroma; these qualitative characteristics influence the consumer and therefore the market demand [3] whose objective is to provide safe beef with high food characteristics.

Both the carcass fat deposition and the meat fatty acid composition play an important role in eating characteristics variation [3]. The World Health Organization [4] recommends a reduction in the intake of saturated fatty acids (SFA) in favor of the n-3 polyunsaturated fatty acids (n-3PUFA), for their beneficial effects on human health. Because of this, the consumer is often led to reduce the presence of meat in the daily diet, forgetting the nutritional value of meat as a source of protein, vitamins, minerals and for its content of bioactive substances such as conjugated linoleic acid isomers (CLA) and branched chain fatty acids [5].

The diet is the main factor affecting the quantity and quality of fat in beef [6]. Nevertheless, the rumen is a formidable barrier to the delivery of unsaturated fatty acids (UFA) to the small intestine. The biohydrogenation of dietary UFA by ruminal microbes enriches duodenal chyme with SFA, which then are absorbed and deposited in body tissues. Saturated fatty acids, whose levels are high in ruminant meat, are one of the main concerns about the health-fullness of beef [6] due to the hypercholesterolemic and thrombogenic effects and the increased risk for thrombosis [7] and cardiovascular disease. Consequently, the objective of several researches [8,9,10] was to study the effect of the diet on the fatty acid profile of the muscle and fat depots in ruminants in order to increase the monounsaturated fatty acids, and particularly the oleic acid that, reducing the oxidation of the cholesterol LDL, may slow the progression of atherosclerosis, and PUFA, for their beneficial effects on the human health [6].

The increasing request by consumers for food that is safe and healthy has focused the researches on feeding strategies such as the dietary supplementation of unsaturated vegetable oils [11,12]. In this context, the supplementation of agricultural industry co-products in animal feeding can be an interesting strategy both to minimize their eco-footprint [13] and to influence the quality of products of animal origin, especially in the case of co-products that contain appreciable quantities of vegetable oils, which may contribute to increase the UFA content of ruminant meat. A survey of the literature found papers dealing with the use of olive cake, the main co-product of the olive oil industry, in dairy cow [14], sheep [15,16], lambs [8], goat [17], pig [18], poultry [19], and rabbit [20] feeding, although scarce literature appears to be available on the use of this co-product in beef cattle feeding [21]. The inclusion of olive cake in ruminant nutrition can negatively affect the digestibility of the organic matter due to the high lignin content [16]. Modern extraction technologies allow, by using a three-phase extraction system with low water addition level, a preventive destoning of the olives, improving the quality of the oil, containing antioxidants, such as tocopherols and retinol and bioactive phenols, and reducing, in all or in part, the presence of the seeds (stoning) and therefore the lignin content [22]. Consequently, olive co-products must be considered as a food resource and not as a waste, able to compete with conventional feeds.

We hypothesized that the intake of a diet containing olive cake would have supplied a “cheap” fiber and energy in beef cattle feeding and a good response on meat in terms of the fatty acid profile.

In this trial, the effect of the dietary partially destoned olive cake supplementation, in two doses, on performance, carcass characteristics, meat quality, and fatty acid profile of intensively finished bulls was evaluated.

## 2. Materials and Methods

### 2.1. Ethical Statement

The animals were managed by trained personnel according to the European Union legislation for the protection of animals used for scientific purposes [23]. Animal experimental procedures were authorized by the Regional Department of Agriculture, Rural Development and Mediterranean Fisheries—Sicilian Region (Dipartimento Regionale Agricoltura—Assessorato Regionale dell’Agricoltura, dello Sviluppo Rurale e della Pesca Mediterranea—Regione Siciliana) Italy, n. 94750023262, prot. 110180.

### 2.2. Animals and Diets

For the trial, a total of 45 Limousin young growing fattening bulls born in the same season, weaned and reared under the same management and feeding systems were chosen. After a 2-week adaptation period, the animals were randomly divided into three homogeneous groups based on the age (11 months) and body weight (440.0 ± 10.5 kg), called CTR (Control), LOC (Low Olive Cake), and HOC (High Olive Cake). Bulls were fattened in the same commercial farm and were housed in 15 contiguous straw pens, each one organized with 3 bulls, with a space allowance of 3 m^2^/head. The three groups were fed twice daily, in the morning and in the afternoon, on formulated diets differing only for the olive cake content. The control group (CTR: 15 animals; five pens) received a concentrate without any dietary olive cake supplementation, whereas the experimental groups, LOC and HOC, received a concentrate supplemented with the dried partially destoned olive cake (DOC, *cv. Menfi)* as 7.5% (LOC: 15 animals; five pens) and 15.0% (HOC: 15 animals; five pens) on a DM (Table 1). The daily intake of dry matter was 2% of each bull’s body weight. The daily diet of each bull was composed of 1.5 kg of concentrate calculated for 100 kg of body weight and it was integrated with wheat straw in the proportion of 75:25 (concentrate/straw) as a total mixed ration (TMR). Twice daily, in the morning and in the afternoon, in the mobile mixer-wagon, the ingredients of each diet (CTR, LOC, and HOC) were separately weighted, mixed with the straw, and distributed as TMR to animals. Three mixed-wagons were prepared each time (six per day). The adaptation period to the diets lasted two weeks. At the beginning of the adaptation period (Day 0) all animals received the control diet (concentrate without any inclusion of olive cake). Then, DOC was gradually added, into the mixer-wagon, to the ingredients of the diets (LOC and HOC), until to the inclusion percentage (LOC diet: 7.5%; HOC diet: 15.0%) was reached (Day 14). This day was considered time zero.

Samples of the concentrate and straw were collected at the beginning, middle and end of the trial. The total amount collected at each time and for each group was mixed, and a final sample was taken for each concentrate and for the straw. After drying, the olive cake was stored in boxes and sampled from the top, middle, and bottom. The total amount collected at each time was pooled, and a final sample was taken to be submitted to the laboratory analyses.

Samples of concentrates, straw and DOC were analyzed for DM (method 930.15 1999), CP (method 2001.11 2005), EE (method 920.39 1920) and ash (method 942.05 1942) according to AOAC International [24]. The total starch was determined by an enzymatic colorimetric method (method 996.11) according to AOAC International [25], with an assay kit from Megazyme International Ltd. (Wicklow, Ireland). The NDF was determined using the method reported by Goering and Van Soest [26]. The proximate composition of the concentrates and straw is reported in Table 1. Table 2 shows the fatty acid composition of the concentrates. Table 3 shows the chemical composition and the fatty acid profile of the DOC.

### 2.3. Animal Monitoring

The trial was conducted for 150 days and all bulls of each pen, before TMR distribution, were individually weighed at the beginning of the trial, after 80 days, and at the end of the trial, to calculate the individual average daily gain (ADG). Upon arrival, bulls were vaccinated, treated for external and internal parasites and, since the beginning of the trial, they were checked by the veterinarian, twice daily, to verify the health status. At the end of the trial, all bulls, on the same day, were humanely slaughtered in an authorized, commercial, EU-licensed abattoir, following the recommendations of the European Council [27] concerning the protection of animals at the time of killing.

As reported in Cozzi et al. [28], around thirty minutes after slaughter, the hot carcass weight was recorded. Then, the carcasses were aged in a refrigerated slaughterhouse room for 7 days at controlled temperature (0–4 °C) and humidity (62–87%).

### 2.4. Technological and Chemical Analyses

To evaluate the meat quality characteristics, seven days after the slaughter, individual samples of *Longissimus dorsi* muscle, between the seventh and eighth thoracic vertebrae included, were removed for the analyses. The pH and temperature were measured by a portable pH-meter provided with a Hamilton double-pore glass-piercing electrode WTW 330/SET 1 (Weilheim, Germany). The meat color was determined according to the CIE L*, a*, and b* system using a portable colorimeter (Konica Minolta CM-600d, Chiyoda, Japan).

The color of the raw meat was measured on a freshly cut surface of slices (20 mm of thickness), after being exposed to the atmospheric oxygen for 30 min [29]. Samples of meat were measured by scanning 5 different locations. The instrument was calibrated with a white standard plate before measurements. The Hue angle (H°) and Chroma (C*) were calculated as [30]:

(tan^−1^ (b*/a*)) and ((a*^2^ +b*^2^)^1/2^).

The cooking loss was determined for each sample of 190 g, by immersing the individual bag in a 95 °C water bath (WB-OD24, FALC Instruments, Treviglio (BG), Italy) for several minutes until the internal temperature reached 75 °C. The sample temperature was detected using a thermocouple thermometer AMA-DIGIT (Buddeberg GmbH, Mannheim, Germany). After cooking and before opening the bags, each sample was tempered at room temperature to drain the liquid. The cooking loss was calculated using the formula:

((weight of raw meat-weight of cooked)/weight of raw meat × 100).

The tenderness of the cooked sample was assessed by determining the shear force, using a Warner-Bratzler (WBSF) cell mounted on an Instron 5542 (Instron, High Wycombe, UK) universal testing machine. The measurement was recorded as the peak yield force in kg/cm^2^, required to shear, at a 200 mm/min crosshead speed, perpendicularly to the direction of the fibers on three cylindrical cross-section (10 mm diameter and 25 mm length) replicates from each sample [30].

On each sample, the moisture, fat and protein were determined according to Anderson [31] and collagen and salt content by using the Near Infrared Spectroscopy in Transmittance (FoodScan^™^ Meat Analyser; FOSS, Padova, Italy).

### 2.5. Fatty Acids of Feed and Total Intramuscular Lipids

The fatty acid methyl esters (FAME) of feeds and the total intramuscular fat (IMF) were analyzed by the GC–FID. Lipids were extracted according to the method described by Folch et al. [32]. The fatty acids methyl esters (FAMEs) were prepared [33] by using a solution of sulfuric acid/methanol (1:9, *v*/*v*).

For FAMEs of feed, a gas chromatograph with a FID detector (Agilent Technologies 6890 N, Palo Alto, CA, USA) equipped with an Omegawax 250 column (Supelco, Bellefonte, PA, USA; 30 m × 0.25 mm i.d., 0.25 μm film thickness) was used. The column temperature was programmed: An initial isotherm of 100 °C (5 min), an increment of 4 °C/min and a final isotherm of 240 °C (20 min). The temperature of the injector and detector was 250 °C. The injection volume was 0.5 μL. The carrier gas used was helium (1 mL/min), and the split ratio was 1:50.

For the IMF, FAMEs were analyzed by a gas chromatograph with a FID detector (Agilent Technologies 6890 N, Palo Alto, CA, USA) equipped with a SP-2560 fused silica capillary column (100 m × 0.25 mm i.d. × 0.2 µm film thickness, Supelco, Inc., Bellefonte, PA, USA). The column temperature was programmed: An initial isotherm of 160 °C (6 min.), an increment of 3 °C/min and a final isotherm of 250 °C (30 min.) [34]. The temperature of the injector and detector: 250 °C. The injection volume: 1.0 μL. The carrier gas used was helium (1 mL/min), and the split ratio was 1:50.

Fatty acids of feed and IMF were identified by comparing the retention times of FAME peaks from samples containing standards from Supelco (mix 37 FAMEs; Supelco, Inc., Bellefonte, PA, USA). Chromatogram peak areas were acquired and calculated using a Chemstation software (Agilent, Inc., Santa Clara, CA, USA). The concentration of each fatty acid was expressed as g/100 g, considering 100 g as the summation of the areas of all FAMEs identified. For each sample, the chromatographic analysis was replicated three times.

The amount of each fatty acid was used to calculate the indices of atherogenicity (AI) and thrombogenicity (TI), as proposed by Ulbricht and Southgate [35], the hypocholesterolaemic/hypercholesterolaemic ratio (HH), as suggested by Santos-Silva et al. [36] and the peroxidation index (PI) as proposed by Luciano et al. [9]:

$$\mathrm{AI}=\frac{C12:0+4\;\left(C14:0\right)+\;C16:0}{\Sigma \;\mathrm{MUFA}\;+\;\Sigma \;n\;-6\mathrm{PUFA}\;+\;\Sigma \;n\;-3\mathrm{PUFA}}$$

$$\mathrm{TI}=\frac{C14:0+\;C16:0+\;C18:0}{\;\left(0.5\times \;\Sigma \;\mathrm{MUFA}\right)+\left(0.5\times \Sigma \;n\;-6\mathrm{PUFA}\right)+\left(3\times \;\Sigma \;n\;-3\mathrm{PUFA}\right)+\left(\Sigma \;n\;-3\mathrm{PUFA}/\Sigma \;n\;-6\mathrm{PUFA}\right)}$$

$$\mathrm{HH}=\frac{C18:1n-9+\;C18:2n-6+\;C20:4n-6+\;C18:3n-3+\;C20:5n-3+\;C22:5n-3+C22:6n-3}{C14:0+\;C16:0}$$

PI=(% dienoic × 1)+(% trienoic × 2)+(% tetraenoic × 3)+(% pentaenoic × 4)+(% hexaenoic × 5)

### 2.6. Statistical Analysis

For performance parameters, pen means served as the experimental unit for statistical analysis. Data were analyzed by a mixed model analysis [37], using diet as the classification factor with the following model:

*Yijkl* = *μ* + *Di* + *Pj* + (*Di* × *Pj*) + *Ak*(*i*) + *εijkl*

where *Yijkl* are the observations, *μ* the overall mean, *Di* the fixed effect of diet *i* (*i* = 3), *Pj* the fixed effect of period *j* (*j* = 3 for BW; *j* = 2 for ADG), *Ak*(*i*) is the random effect of pen *k* (*k* = 15) nested within diet *i* and *εijkl* is the random residual. The interaction between the two fixed effects was tested and removed because it was not significant; therefore, only the fixed effects were discussed.

Data of the carcass and meat quality were submitted to a mixed model analysis [37], which included the effect of the diet (CTR, LOC, HOC) as fixed factor and the pen as random factor. Least square means (LSM) and standard error of least square means (SEM) were calculated. Comparisons between LS means were performed using the Tukey test. Differences were considered significant at *p* < 0.05.

## 3. Results

### 3.1. Animal Performance and Carcass Characteristics

Both levels of the olive cake inclusion in the diets increased the body weight and ADG, showing higher (*p* < 0.05) values in bulls from LOC and HOC groups compared to that from CTR group. As expected, the body weight of bulls increased progressively (*p* < 0.05) in relation to the period, reaching the highest value at slaughter (Table 4). A higher (*p* < 0.05) growth rate value was observed at the 80th day of the trial compared to that at the 150th day of the trial. The growth rate, calculated from the beginning (Day 0) to the end (Day 150) of the trial, showed a lower (*p* < 0.05) value compared to that observed in the 1st period and a higher (*p* < 0.05) value than that observed in the 2nd period.

### 3.2. Meat Characteristics

Carcass weights and dressing percentages were higher (*p* < 0.05) in LOC and HOC groups than those of the CTR group, whereas they did not show significant (*p* > 0.05) differences between the bulls from HOC and LOC groups (Table 5).

The pH, L*, and a* values and Chroma were not affected (*p* > 0.05) by the dietary olive cake supplementation. On the other hand, the b* values were similar (*p* > 0.05) between LOC and HOC groups, but higher (*p* < 0.05) compared to that of the CTR (Table 5). H° value increased (*p* < 0.05) in relation to the dose of the dietary olive cake supplementation (Table 5). The cooking loss and shear force showed the significant (*p* < 0.05) highest values in the HOC group compared to those of the CTR and LOC groups (Table 5).

As regards the chemical composition of *Longissimus lumborum* muscles, no differences (*p* > 0.05) were observed for water and protein contents among the groups, while the IMF content increased (*p* < 0.05) in relation to the levels of the dietary olive cake supplementation (Table 5).

### 3.3. Fatty Acids in Dried Partially Destoned Olive Cake and Concentrates

The oleic acid (C18:1n-9) was the most abundant fatty acid in the LOC and HOC concentrates (Table 2). The palmitic (C16:0) and linoleic (C18:2n-6) acids were the other acids that were also present, whereas the stearic (C18:0) and linolenic (C18:3n-3) acids were the fatty acids less present in LOC and HOC concentrates. LOC and HOC concentrates showed a higher content of the oleic acid and a lower linoleic and linolenic acid percentages compared to CTR concentrate. Consequently, the MUFA showed a higher content and the PUFA a lower content in the LOC and HOC concentrates compared to those of the CTR concentrate (Table 2). The UFA/SFA ratio increased with the inclusion of the olive cake (Table 2).

### 3.4. Fatty Acids Composition of Total Intramuscular Lipids

Among the fatty acids of nutritional interest, some changes involved the palmitic, palmitoleic and CLA isomer (C18:2 *cis*-9, *trans*-11) acids, which showed (*p* < 0.05) lower levels in the LOC and HOC groups compared to those of the CTR group; whereas, the stearic, oleic, *trans* isomers of oleic (C18:1 *trans*-11 and *trans*-10), alfa-linolenic, arachidonic and eicosapentaenoic acids showed higher (*p* < 0.05) levels in the LOC and HOC groups compared to those of the CTR group (Table 6).

The supplementation with the DOC caused an increase (*p* < 0.05) in the MUFA and PUFA, both n-6 and n-3 PUFA, and a decrease (*p* < 0.05) in the SFA (Table 7). The UFA/SFA and PUFA/SFA ratios were higher (*p* < 0.05) in the IMF of the HOC group compared to those of CTR and LOC groups. The n6/n3 ratio was lower (*p* < 0.05) in the HOC group than that of the CTR and LOC groups (Table 7). As regards the nutritional indices, the AI and TI were lower (*p* < 0.05) in LOC and HOC groups compared to those of the CTR group (Table 7). Finally, the H/H was higher (*p* < 0.05) in the LOC and HOC groups compared to that of the CTR group. The peroxidation index (PI) was higher (*p* < 0.05) in the HOC group compared to that of CTR and LOC groups (Table 7).

## 4. Discussion

The young bulls fed with diets containing olive cake, showed a better body weight, ADG and carcass characteristics. This could be due to a better utilization of the energetic quota, provided by lipids coming from the olive cake [15]. Probably, the high ADL content of the DOC caused an increase of the rumen transit speed and a partial inactivity of rumen specific microorganisms, involved in the stearic synthesis too [15,16].

The pH values were not influenced by the dietary supplementation of olive cake as observed by Ozdogan et al. [38] in lambs fed with diets including different levels of olive cake. According to Czyzak-Runowska et al. [39] an abnormally high final muscle pH (>6.0) results in the production of a dark-cutting or dark, firm, and dry (DFD) meat. Pipek et al. [40] reported that a meat of high quality has ultimate pH at the range of 5.4–5.6. At pH > 5.8 a decrease in meat delicacy, as well as a possibility of maintaining good quality during cooling, is observed. High pH is improper for sorting, confectioning, and vacuum packaging of the meat. Moreover, high ultimate pH meat can be characterized by gummy structure, increased water-holding capacity and decreased specific taste [40]. The pH values recorded in all groups were within the optimal range indicating a meat of high quality and, therefore, a good acidification of beef.

As regards the instrumental color variables (L*, a*, b*, H° and C*), the values of L* and b* are similar to those observed by Salami et al. [41] in beef from steers fed with grass silage and a concentrate supplement in which barley was substituted with increasing levels of dried citrus pulp whereas, the H° values, that represents perceived meat color, with larger angles indicating a less red product, were lower than those reported by the same Authors. The data confirm that the surface meat discoloration increased progressively with the level of olive cake inclusion in the diet that can be, partially, explained by the increase of the IMF content in the LOC and HOC groups, according to Humada et al. [42].

The values of a* = 12 and C* = 16 are considered as thresholds for visual discoloration that limits consumer acceptability of beef [43]. The current data indicated that values of a* (redness) and C* (color vividness) measured in all groups were greater than the threshold values, suggesting a good visual acceptability of beef.

As regards the cooking losses and tenderness, it is generally accepted [44] that the IMF positively influences the tenderness and the overall acceptability of meat in different species because of very low levels of the IMF lead to a dry and less-tasty meat. However, significant correlations with technological quality traits are often observed when there is a high variation in the IMF content. In our study, the IMF was 60% higher in the HOC group compared to the CTR group, highlighting a more tender meat with a less weight loss after the cooking than that of the CTR group. Moreover, Mwangi et al. [3] report that the soft fat and the palatability of beef are associated with a high concentration of oleic acid in the IMF, as found in our results.

The higher values of the IMF found in this study in the LOC and HOC groups can be due to the higher fat content of the diets containing the olive cake compared to that of the CTR diet. According to Silva et al. [45], the partial grains’ substitution for fats and oils in the ruminants’ feed increases energy density and nutritional efficiency.

The dried olive cake (DOC) consisted of up to 80% of oleic acid (C18:1 *c9*), along with the palmitic (C16:0) and linoleic (C18:2n-6) acids [46]. Small traces of the stearic (C18:0) and α-linolenic (C18:3n-3) acids have been found in the olive cake such as in the pomace and olive residue [46].

The fatty acid profile of meat has a great impact on meat quality, sensorial characteristics, consumer acceptance, and human health [47]. In this study, the dietary olive cake supplementation in bull diets affected the intramuscular (IM) fatty acid profile.

The oleic acid was influenced by the increasing inclusion levels of the DOC in bull diets, showing a higher content in the total IMF of the HOC group. This increase could be due to the high proportion of the oleic acid in DOC changed remarkably its content into the diets (LOC: 43.0% and HOC: 46.8%) given to bulls. Our results agree with several reports providing evidence that the dietary supplementation of ruminants with vegetable oils increases the proportion of UFA in the muscle [11,12]. In particular, the dietary administration of the DOC could be proposed as a strategy to increase the levels of some desirable fatty acids [14]. Data are not in agreement with Kotsampasi et al. [8] that found no differences in the oleic acid content of the IMF of lambs fed diets including from 80 to 240 g/kg of the partly destoned exhausted olive cake; this could be due to the lower level of oleic acid in the destoned olive cake (39.2 g/100 g of total FA) used by Kotsampasi et al. [8] than that used in this trial (67.2 g/100 g of total fatty acids) and consequently to the low levels of oleic acid into the diets supplied to lambs.

According to Mele et al. [11], the lower rumenic acid (RA – C18:2 *c*-9, *t*-11 CLA) levels in the LOC and HOC groups could be associated to the low linoleic level (C18:2n-6 – LA) in the LOC and HOC diets than that of the CTR diet (32.0% and 30.0% vs. 51.9%) and, consequently, to the low lipolysis and biohydrogenation processes, during which the RA is formed (isomerization) as intermediates during biohydrogenation of the LA [48] to trans-vaccenic (TVA – C18:1 *t11*) and hence to stearic acid. Another explanation could be that the TVA produced in the rumen, through the blood stream, reaches the lower gut and is incorporated in the muscular tissue where the Δ9 desaturase converts the TVA to its corresponding conjugated linoleic acid (CLA) isomer, the RA ([49]. Previously, it was believed that the CLA produced in the rumen was the most important source of the CLA in the milk and meat, but studies strongly suggest that the endogenous synthesis of the RA in the mammary gland [50] or in the subcutaneous or in the IMF [51], is the predominant production pathway. Thus, the highest CLA and the lowest TVA proportion in muscle of animals fed CTR diet could suggest that the Δ9 desaturase was more active in CTR animals, and consequently, more TVA was converted into CLA in tissues.

These changes were reflected in the tissue fatty acid compositions and could confirm our data. Interestingly, TVA and RA have exhibited potent biological activity with anti-carcinogenic and hypo-lipidemic properties in cell cultures and animal models [6].

In the IMF, the content of *trans* monoenes was higher in the LOC and HOC groups than those of the CTR group, as well as the content of the stearic acid, suggesting an intensive biohydrogenation of the higher dietary oleic acid content [11] in the LOC and HOC diets than that of the CTR diet (43.0% and 46.8% vs. 22.9%). This relation was seen previously by Mosley et al. [47], who stated that the biohydrogenation of the C18:1 *c9* by mixed ruminal microbes involves the formation of several positional isomers of *trans* monoenes, as well as the contributing to the direct biohydrogenation to C18:0. The C18:1 *t10* was the major isomer, representing about 77% of total trans-18:1 fatty isomers in the IMF of the CTR, LOC and HOC meat. A high level of the C18:1 *t10* in the IMF have been previously reported in intensively reared lambs, especially when the UFA has been added to the diet [52]. According to the pattern of the *trans*—18:1, the biohydrogenation of the oleic acid contained in the LOC and HOC diets resulted in an accumulation of the C18:1 *t10* and C18:1 *c9* [11]. In contrast to C18:1 *c9*, that has beneficial human health effects including anti-inflammatory, anti-carcinogenic, anti-atherosclerotic and anti-diabetic effects, the studies on animal models have shown that the C18:1 *t10* leads to adverse effects on cardiovascular health, similar to that of partially hydrogenated vegetable oils, while, the studies in humans are lacking and the potential long-term effects of C18:1 *t10* intake from consuming ruminant meat on the risk of cardiovascular disease is unknown [53].

The C18:1 *t11*/C18:1 *t10* ratio was not influenced (*p* > 0.05) by the increased levels of dietary olive cake supplementation, showing values (CTR: 0.31; LOC: 0.32; HOC:0.31) similar to those reported by Mapiye et al. [54], in intramuscular fat of steers fed a barley-based diet supplemented with increasing levels of vitamin E.

The LA, the most abundant PUFA in beef, was not significantly affected by the DOC incorporation into the diets. The LA, more represented in the CTR than LOC and HOC diets, may have been subjected to isomerization of the *cis* double bond at carbon 12 to a *trans* double bond at carbon 11 [6], resulting in a higher production of the RA, as described above. This could confirm the higher level of RA in CTR group compared to that observed in LOC and HOC groups.

As regards the α-linolenic acid (C18:3n3- LNA), Dewhurst et al. [55] affirmed that even though the biohydrogenation of LNA is 86 to 94% in the rumen, when the olive cake is added to the diet, nearly 2.4 times more α-linolenic is able to escape from the rumen due to the polyphenol oxidase (PPO) activity of the olive cake [56]. This could confirm the higher LNA content detected in the IMF of beef fed with LOC and HOC concentrates. Moreover, it is also possible that the α-linolenic acid escapes the biohydrogenation to stearic, for the high level of the oleic acid, in LOC and HOC groups, as a substrate to the synthesis of the stearic acid [57]. Indeed, in beef cattle, the oleic acid is hydrogenated largely to stearic acid by ruminal microorganisms [57].

The muscle contains a significant proportion of the long chain (C20-22) PUFA which originates in meat either directly from the feed or from the desaturation and elongation of the LNA and the LA by the Δ5 and ∆6 desaturases [58]. Dietary fatty acids and the type of biological tissue are some of the different factors that can influence the activity of these desaturases [59].

The arachidonic acid (20:4n-6, ARA) and the eicosapentaenoic acid (C20:5n-3, EPA) have various metabolic roles including the eicosanoid production [60]. It is interesting to observe, that the higher levels of the EPA and ARA in the intramuscular fat of the HOC group than those of the CTR and LOC groups.

In addition to the LNA and EPA, beef naturally contains a low level of the n-3 fatty acids such as the docosapentaenenoic (C22:5n-3, DPA) and the docosahexaenoic (C22:6n-3, DHA) acids which are, together with the EPA, long chain elongation and desaturation products of LNA. The consumption of n-3 fatty acids, mainly contained in the fish oil (i.e., EPA and DHA), leads to many positive physiological and behavioral effects, including those on cognition in humans [61]. In this direction, many studies have tried to fix the reference dietary intakes for these fatty acids [62]. The DPA is more common than the DHA in beef because of the desaturation/elongation chain of n-3 fatty acids, from the LNA to DHA, seems to block at the level of the DPA [63]. Consequently, in populations where little or no oily fish is consumed, beef can still be an important source of long chain n-3 fatty acids, particularly when the DPA is included [64]. In this trial, the DPA and DHA showed slightly higher values in the IMF of the HOC group than that of the CTR and LOC groups.

As regards the fatty acid classes, the higher levels of the MUFA, especially of the oleic acid, in muscles of the HOC group, reducing the oxidation of the cholesterol LDL and the blood pressure, may slow the progression of atherosclerosis [7].

The PUFA, that show the highest level in the HOC diet, are preferential substrates for the onset and propagation of the lipid oxidation in the muscle. However, an increase in the concentration of the UFA can have deleterious effects on the oxidative stability of meat and, in this context, it is important to consider the overall degree of unsaturation of the IMF fatty acids, as their susceptibility to oxidation increases with increasing unsaturation degree and, consequently, the potentially adverse health effects of their lipoperoxidation products [15]. In our samples, the peroxidation index (PI) values were significantly affected by the dietary treatments and increased progressively with the level of olive cake inclusion in the diet. In summary, the inclusion of the DOC in the diet affected the fatty acid composition of meat and increased its susceptibility to oxidative processes [9]. Nevertheless, the oxidative stability could be in part offset by the polyphenol content of the olive cake. The olive pomace has been widely accepted as one of the highest antioxidants, thanks to the presence of some important antioxidants and phenolic compounds (oleuropein, verbascoside, ligstroside, lutein, apigenine, tyrosol, and hydroxytyrosol) [65]. Several publications reported that the remarkable olive oil resistance to the oxidation was closely linked to its total polyphenol content [65]. From the existing information, we could infer that crude extracts of olive polyphenols may provide good protection against the lipid oxidation and the off-flavor development in pre-cooked meat. Besides, Abdalla et al. [66] report that the antioxidant compounds of the olive pomace can be used to improve the negative effect of the heat stress especially, oxidative enzymes, some blood constituents and growth performance in heat stressed calves.

An unbalanced n6/n3 ratio in diets may increase the risk of cancers and coronary heart diseases [4]. The studies on the relationship between the n-6/n-3 ratio and the pathogenesis of many diseases, including cancer, cardiovascular, inflammatory and autoimmune diseases, indicate that the optimal ratio in ruminant meat, for the World Health Organization [4], is less than 4. In the present study, n6/n3 ratio ranged from 3.21 (HOC group) to 3.49 (CTR group), within the recommended values, and in line with the values reported by Kotsampasi et al. [8] whereas, lower enough from those reported by Mele et al. [11] and Vasta et al. [67].

As regards the nutritional indices, the atherogenic (AI) and thrombogenic (TI) indices might better characterize the health properties of vegetables or animal food than a simple approach based on total SFA content or on the ratio PUFA/SFA [68]. Specifically, AI and TI applied by Ulbright and Southgate [35] indicate the relationship between the sum of three SFA (AI = C12:0, C14:0, C16:0; TI = C14:0, C16:0, C18:0), which are hypercholesterolemic, and the sum of the main classes of UFA, which are antiatherogenic and antithrombogenic. Therefore, AI and TI are considered indicators or measurements of the level of the atherogenicity and thrombogenicity. Our data show the lowest, therefore, the best atherogenic and thrombogenic indices in the HOC group.

Finally, in addition to the evaluation of the atherogenic and thrombogenic indices, a good approach to the nutritional evaluation of the fat should be the utilization of another index based on functional effects of fatty acids, the ratio hypocholesterolaemic:hypercholesterolaemic fatty acids (HH), computed according to present knowledge of the effects of individual fatty acids on the cholesterol metabolism [69]. The highest values observed for the HH index of the HOC group, together with the atherogenic and thrombogenic indices, confirm the better nutritional value of beef of the HOC group.

## 5. Conclusions

The results confirm the validity of our hypothesis: the dietary inclusion of partially destoned olive cake influenced the technological parameters, intramuscular fat content and quality indices of beef without compromise the in vivo animal performance. Moreover, data show a “dose–effect” of the olive cake as confirmed by the impact of the highest supplementation on some meat quality traits parameters, some fatty acids of nutritional interest (CLA, ARA, EPA), fatty acid classes (SFA, MUFA, PUFA), quality indices (AI, H/H) and the peroxidation index.

Therefore, the use of the olive cake in substitution to a quote of cereals in the diet of beef could be a strategy for an efficient reutilization of olive oil industry residues, to reduce the environmental impact of the food production, to increase the sustainability of rural economies and to improve the agriculture profitability. It allows us to turn a low-quality material into a high-quality food, introducing into the diet a source of oleic acid and some substances with nutraceutical properties, which could contribute to sustain the animal performance as well as to obtain a good meat quality.

In conclusion, according to our observations, olive cake can be considered as a functional component in beef.

## Figures and Tables

**Table 1:** Ingredients and proximate composition of the concentrates and straw.

Item	Concentrates			Straw
	CTR	LOC	HOC	
Ingredients (% of DM)				
Corn	45	44.7	37	
Soybean meal, 44	18	18	12	
Barley	15	10	18	
Olive cake	0	7.5	15	
Wheat bran	11	7.5	5	
Sunflower	2	2.3	4	
Molasses	2	2.5	2	
Carob pulp	2	2	2	
Vitamin and mineral mix ^§^	5	5.5	5	
Proximate composition				
DM (%)	89.15	88.12	88.22	88.00
CP (% of DM)	15.11	15.06	15.01	3.70
EE (% of DM)	3.51	5.14	7.30	2.20
NDF (% of DM)	11.02	16.08	18.05	79.80
Starch (% of DM)	38.44	36.94	34.06	1.90
Net Energy (UFV kg^−1^ DM) ^¶^	0.91	0.93	0.95	0.30

^§^ Providing per kg of diet: 8000 U vitamin A, 1200 U Vitamin D_3_, 20 mg Vitamin E, 85.6 mg Manganese sulfate, monohydrate, 76.7 mg Zinc sulfate monohydrate, 54.6 mg Zinc oxide, 11.8 mg Copper sulfate pentahydrate, 0.5 mg Anhydrous calcium iodate, 0.4 mg Sodium selenite; ^¶^ UFV: from the French “Unité Fourragère Viande” = feed unit for meat. Concentrate: CTR, no inclusion of olive cake; LOC, inclusion of 75 g/kg of olive cake; HOC, inclusion of 150 g/kg of olive cake; DM = Dry Matter; CP = Crude Protein; EE = Ether Extract; NDF = Neutral Detergent Fiber.

**Table 2:** Fatty acids of nutritional interest, fatty acid classes (g/100 g fatty acid methyl esters (FAME)) ^#^ and UFA/SFA ratio of the concentrates.

Diet	CTR	LOC	HOC
Fatty acid profile			
C14:0	0.26	0.23	0.18
C16:0	16.18	15.10	14.65
C18:0	3.08	2.70	2.40
C18:1n9	22.94	43.02	46.81
C18:2n6	51.91	32.04	30.01
C18:3n3	3.50	1.88	1.62
Fatty acid classes			
SFA	19.85	19.50	18.66
MUFA	23.96	44.67	49.04
PUFA	56.19	35.83	32.30
n-3 PUFA	3.80	2.03	1.84
n-6 UFA	52.30	33.70	30.38
Fatty acid ratio			
UFA/SFA ratio	4.04	4.13	4.36

^#^ The concentration of fatty acid was expressed as g/100 g, considering 100 g the sum of the areas of all FAME identified. Concentrate: CTR, no inclusion of olive cake; LOC, inclusion of 75 g/kg of olive cake; HOC, inclusion of 150 g/kg of olive cake; SFA = saturated fatty acids; MUFA = monounsaturated fatty acids; PUFA = polyunsaturated fatty acids. USFA/SFA = unsaturated fatty acids/saturated fatty acids ratio. C14:0 = Myristic acid; C16:0 = Palmitic acid; C18:0 = Stearic acid; C18:1n9 = Oleic acid; C18:2n6 = Linoleic acid; C18:3n3 = alfa-Linolenic acid.

**Table 3:** Chemical and acidic composition of the dried olive cake used in the trial.

Dried Olive Cake	Item
Chemical composition (g/kg as fed)	
Moisture	43.9
Crude protein	86.3
Ether extract	303.4
Neutral detergent fiber	493.7
Acid detergent fiber	393.9
Acid detergent lignin	230.6
Ash	40.9
Starch	14.8
Fatty acids (g/100 g of FAME) ^#^	
C14:0	1.76
C16:0	14.43
C18:0	3.52
C18:1n-9	67.18
C18:2n-6	8.39
C18:3n-3	0.52

^#^ The concentration of fatty acid was expressed as g/100 g, considering 100 g the sum of the areas of all FAME identified; C14:0 = Myristic acid; C16:0 = Palmitic acid; C18:0 = Stearic acid; C18:1n9 = Oleic acid; C18:2n6 = Linoleic acid; C18:3n3 = alfa-Linolenic acid.

**Table 4:** Effect of dietary olive cake supplementation on in vivo performance of Limousin young bull during the trial.

	Diet ^¥^			SEM	Period ^§^			SEM	*p*-Value ^‡^	
	CTR	LOC	HOC		Initial	Intermediate	At Slaughter		Diet	Period
BW, kg	553 ^b^	589 ^a^	588 ^a^	1.28	446.30 ^c^	581.80 ^b^	686.50 ^a^	1.44	0.018	0.032
	CTR	LOC	HOC		1st period	2nd period	Total			
ADG, kg/d	1.43 ^b^	1.88 ^a^	1.86 ^a^	0.08	1.73 ^a^	1.51 ^c^	1.63 ^b^	0.87	0.042	0.038

^¥^ Dietary treatments: CTR group: fed concentrate without olive cake supplementations; LOC: fed concentrate with 75 g/kg of olive cake; HOC: fed concentrate with 150 g/kg of olive cake. ^§^ Period for body weight: Initial: Day 0; Intermediate: Day 80; At slaughter: Day 150. ^§^ Period for growth rate: 1st period: 0–80 days; 2nd period: 81–150 days; Total: 0–150 days; SEM = standard error of least square means. ^‡^ Probability values of the body weight and Growth rate for the effects of Diet, Period; ^a,b,c^ Within rows, means with different superscripts letter were significantly different (*p* < 0.05); BW = Body Weight; ADG = Average Daily gain.

**Table 5:** Effect of dietary olive cake supplementation on carcass traits and physicochemical properties of *Longissimus lumborum* muscle at 7 days of storage of Limousin young bull.

Item	Dietary Treatments ^¥^			SEM	*p*-Values
	CTR	LOC	HOC		
No. of animals/samplings	15	15	15		
Weight, kg	427 ^b^	488 ^a^	490 ^a^	17.7	0.038
Dressing, %	65.08 ^b^	68.24 ^a^	68.18 ^a^	1.15	0.045
pH	5.53	5.54	5.52	0.12	0.159
Lightness (L*)	45.94	48.27	46.95	3.45	0.228
Redness (a*)	23.42	20.55	20.88	1.80	0.058
Yellowness (b*)	8.68 ^b^	14.01 ^a^	15.99 ^a^	1.50	0.037
Hue angle (H°)	20.34 ^c^	34.28 ^b^	37.45 ^a^	3.10	0.015
Chroma (C*)	24.98	24.87	26.30	8.15	0.087
Cooking loss (%)	35.14 ^a^	33.77 ^a^	30.82 ^b^	1.95	0.045
Shear force (km/cm^2^)	2.26 ^a^	2.13 ^a^	1.82 ^b^	0.11	0.018
Water content (g/100 g muscle)	74.73	74.85	74.81	3.11	0.231
IMF content (g/100 g muscle)	1.16 ^c^	1.63 ^b^	1.87 ^a^	0.09	0.035
Protein content (g/100 g muscle)	20.59	21.14	21.85	1.11	0.092

^¥^ Dietary treatments: CTR group: fed concentrate without olive cake supplementations; LOC: fed concentrate with 75 g/kg of olive cake; HOC: fed concentrate with 150 g/kg of olive cake. IMF = Intramuscular Fat. ^a,b,c^ Mean values with different letters in superscript within rows indicate significant differences (*p* < 0.05); SEM = standard error of least square means. Each sample of muscle was analyzed in triplicate.

**Table 6:** Effect of dietary olive cake supplementation on fatty acid profile (g/100 g) ^§^ of total lipids in the *Longissimus lumborum* muscle of Limousin young bull.

Item	Dietary Treatments ^¥^				
	CTR	LOC	HOC	SEM	*p*-Values
C14:0	2.77	2.62	2.58	0.44	0.219
C15:0	0.51	0.48	0.46	0.03	0.101
C16:0	26.77 ^a^	23.47 ^b^	20.08 ^c^	1.50	0.001
C16:1	2.75 ^a^	1.89 ^b^	1.97 ^b^	0.20	0.001
C17:0	1.22 ^a^	1.28 ^a^	0.89 ^b^	0.12	0.002
C17:1	0.62 ^a^	0.41 ^b^	0.35 ^b^	0.03	0.001
C18:0	15.47 ^b^	17.43 ^a^	19.29 ^a^	2.98	0.041
C18:1 *c9*	37.64 ^c^	39.14 ^b^	40.39 ^a^	5.44	0.038
C18:1 *t10*	2.81 ^b^	3.13 ^a^	3.32 ^a^	0.30	0.045
C18:1 *t11*	0.85 ^b^	1.01 ^a^	1.02 ^a^	0.22	0.046
C18:2n-6	4.84	5.00	5.15	0.30	0.051
C18:2 *c9, t11*	0.39 ^a^	0.34 ^b^	0.30 ^c^	0.01	0.028
C18:3n-3	0.72 ^b^	0.85 ^a^	0.92 ^a^	0.06	0.002
C20:0	0.49	0.66	0.66	0.19	0.061
C20:4n-6	1.16 ^c^	1.26 ^b^	1.47 ^a^	0.39	0.042
C20:5n-3	0.37 ^b^	0.41 ^b^	0.47 ^a^	0.08	0.023
C22:5n-3	0.47	0.45	0.49	0.05	0.092
C22:6n-3	0.16	0.18	0.19	0.03	0.054

^§^ The concentration of fatty acids was expressed as g/100 g, considering 100 g the sum of the areas of all identified FAMEs. ^¥^ Dietary treatments: CTR group: fed concentrate without olive cake supplementations; LOC: fed concentrate with 75 g/kg of olive cake; HOC: fed concentrate with 150 g/kg of olive cake ^a,b,c^ Mean values with different letters in superscript within rows indicate significant differences (*p* < 0.05); SEM = standard error of least square means.

**Table 7:** Effect of dietary olive cake supplementation on fatty acid classes (g/100 g) ^§^, ratios and nutritional indices of total lipids in the *Longissimus lumborum* muscle of Limousin young bull.

Item	Dietary Treatments ^¥^				
	CTR	LOC	HOC	SEM	*p*-Values
SFA	47.23 ^a^	45.95 ^a^	43.96 ^b^	5.77	0.006
MUFA	44.66 ^b^	45.56 ^b^	47.05 ^a^	5.94	0.005
PUFA	8.11 ^b^	8.48 ^b^	8.99 ^a^	1.21	0.002
PUFA n3	1.72 ^c^	1.89 ^b^	2.07 ^a^	0.18	0.014
PUFA n6	6.00 ^b^	6.26 ^ab^	6.62 ^a^	0.15	0.002
UFA:SFA ratio	1.12 ^b^	1.18 ^b^	1.27 ^a^	0.03	0.006
PUFA:SFA ratio	0.17 ^b^	0.18 ^b^	0.20 ^a^	0.01	0.023
n6:n3 ratio	3.49 ^a^	3.32 ^ab^	3.21 ^b^	0.44	0.025
AI	0.72 ^a^	0.63 ^b^	0.55 ^c^	0.02	0.001
TI	1.46 ^a^	1.37 ^b^	1.26 ^c^	0.11	0.017
H:H ratio	1.54 ^c^	1.81 ^b^	2.17 ^a^	0.55	0.001
PI	14.33 ^b^	15.14 ^b^	16.48 ^a^	1.88	0.008

^§^ The concentration of fatty acids was expressed as g/100 g, considering 100 g the sum of the areas of all identified FAMEs. ^¥^ Dietary treatments: CTR group: fed concentrate without olive cake supplementations; LOC: fed concentrate with 75 g/kg of olive cake; HOC: fed concentrate with 150 g/kg of olive cake ^a,b,c^ Mean values with different letters in superscript within rows indicate significant differences (*p* < 0.05). SEM, standard error of least square means. AI = atherogenic index; TI = trombogenic index; H:H = hypocholesterolaemic:hypercholesterolaemic ratio; PI = peroxidation index; SFA = Saturated fatty acids; MUFA = Monounsaturated fatty acids; PUFA = Polyunsaturated fatty acids.
